# Engineering a light-controllable fluorescent protein for peroxynitrite detection *via* genetic code expansion

**DOI:** 10.1039/d6cb00083e

**Published:** 2026-06-18

**Authors:** Gloricelly M. Roman Arocho, Parthasarathi Das, Sachin C. Tennakoon, Jared B. Shaw, Wei Niu, Jiantao Guo

**Affiliations:** a Department of Chemistry, University of Nebraska – Lincoln Lincoln NE 68588 USA; b Department of Chemical & Biomolecular Engineering, University of Nebraska-Lincoln Lincoln Nebraska 68588 USA; c Nebraska Center for Integrated Biomolecular Communication, University of Nebraska-Lincoln Lincoln Nebraska 68588 USA jguo4@unl.edu wniu2@unl.edu

## Abstract

We report a noncanonical amino acid-containing photoconvertible fluorescent protein for selective peroxynitrite detection. Incorporation of *p*-borono-l-phenylalanine into circularly permuted mEos2 affords a peroxynitrite-responsive sensor that retains photoconversion capability, expanding chemical diversity in light-controllable fluorescent proteins.

## Abstract 2

A boronate-containing photoswitchable fluorescent protein enables selective peroxynitrite detection while maintaining photoconversion functionality.

## Graphical abstract

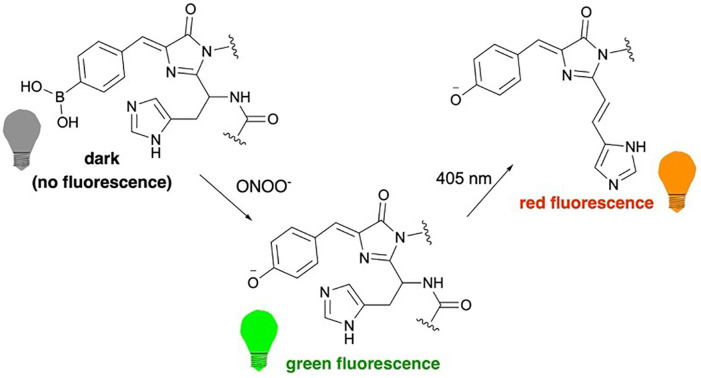

Reactive oxygen and nitrogen species (ROS/RNS) regulate diverse physiological processes yet contribute to disease progression when dysregulated.^1^ Among these species, peroxynitrite (ONOO^−^), generated by diffusion-controlled reaction between nitric oxide (^•^NO) and superoxide (O_2_^•−^),^2^ plays critical roles in cardiovascular, inflammatory and neurodegenerative disorders.^3,4^ Owing to its short half-life (<10 ms) and high reactivity,^5^ direct visualization of peroxynitrite in biological systems remains challenging.

Fluorescent protein (FP)-based biosensors offer genetic encodability and subcellular targeting advantages over small-molecule probes.^6,7^ Boronate-containing chromophores have proven particularly effective for ROS detection due to selective oxidative conversion to phenols.^8–10^ Incorporation of *p*-borono-l-phenylalanine (BoPhe) into FP variants has yielded hydrogen peroxide and ONOO^−^ sensors,^8,11–13^ yet integration of such reactive chemistry into light-controllable fluorescent proteins remains unexplored. Light-controllable FPs such as mEos3.2 undergo irreversible green-to-red photoconversion upon 405 nm irradiation *via* chromophore β-elimination.^14^ The capacity to combine chemical sensing with optical conversion would potentially provide a unique platform for superresolution-compatible imaging.

Here we report the engineering of a photoconvertible peroxynitrite sensor by replacing the chromophore-forming tyrosine of circularly permuted mEos2 (cpmEos2; Fig. 1) with BoPhe. Circular permutation increases chromophore accessibility by repositioning termini and introducing structural openings,^15^ thereby facilitating analyte access to the β-barrel-embedded chromophore.

**Fig. 1:**
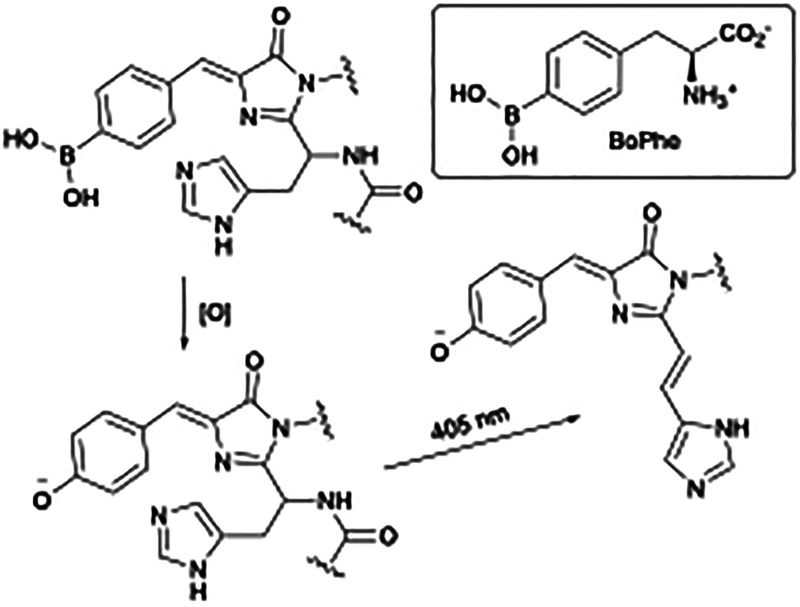
Design of a photoconvertible peroxynitrite-responsive fluorescent protein (FP). Schematic illustration of the sensing and photoconversion mechanism of cpmEos2-Y155BoPhe. Incorporation of *p*-borono-l-phenylalanine (BoPhe) into the chromophore-forming residue generates a boronate-containing chromophore with diminished fluorescence. Oxidation by reactive oxygen/nitrogen species (*e.g.*, peroxynitrite) converts the aryl boronate into a phenolate, restoring the canonical green fluorescent chromophore. Subsequent irradiation with 405 nm light induces the characteristic β-elimination and π-extension reaction of Eos-family fluorescent proteins, yielding the red-emitting Kaede-like chromophore.

Using an orthogonal aminoacyl-tRNA synthetase/tRNA pair,^16^ BoPhe was genetically incorporated at position Y155 (equivalent to Y66 in GFP numbering) of cpmEos2 to afford cpmEos2-Y155BoPhe. The fidelity of the reported aminoacyl-tRNA synthetase/tRNA pair^16^ was sufficient according to our protein expression data (SI, Fig. S1). In the presence of BoPhe, a high level of protein expression was observed. In contrast, only basal expression was detected by western blot in the absence of BoPhe. This result is consistent with previous studies on engineering fluorescent protein mutants containing BoPhe for peroxynitrite detection.^11–13^ The cpmEos2-Y155BoPhe mutant was purified by affinity chromatography (SI, Fig. S2). The protein yield is moderate, reaching approximately 30% of that of wild-type cpmEos2. The incorporation of BoPhe was confirmed by mass spectrometric analysis (calculated mass = 27481.5170, after loss of the N-terminal methionine; observed mass = 27481.7366; SI, Fig. S3).

The purified cpmEos2-Y155BoPhe protein exhibited low basal fluorescence, consistent with electron-deficient chromophore formation caused by boronate substitution.^8^ Mixing 200 µL cpmEos2-Y155BoPhe (3.9 µM) with 50 µL of peroxynitrite (50 µM) at room temperature resulted in a ∼5-fold emission increase after 10 min of incubation (*λ*_ex_ = 485 nm, *λ*_em_ = 530 nm; Fig. 2A). The peroxynitrite-treated cpmEos2-Y155BoPhe exhibited a fluorescence emission profile that resembles that of wild-type cpmEos2, but with reduced intensity and slight spectral broadening (SI, Fig. S4). Both samples were prepared at the same initial volume (250 µL) and protein concentration (3.9 µM). The incomplete recovery of fluorescence suggests that oxidation of the boronate group was not fully complete under the conditions used. The observed spectral broadening likely reflects heterogeneity in the chromophore environment following peroxynitrite treatment, potentially arising from partial oxidation of residues (*e.g.*, cysteine or methionine) within the β-barrel surrounding the chromophore. This was confirmed by mass spectrometric analysis, which revealed multiple species corresponding to different extents of oxidation of the three cysteine and eight methionine residues in the protein following peroxynitrite treatment (SI, Fig. S5). Such modifications could perturb the local chromophore environment and conformational dynamics, thereby contributing to the reduced fluorescence intensity and broadened emission spectra relative to wild-type cpmEos2. Nevertheless, the substantial recovery of fluorescence and preservation of spectral features demonstrate effective activation of the sensor.

**Fig. 2:**
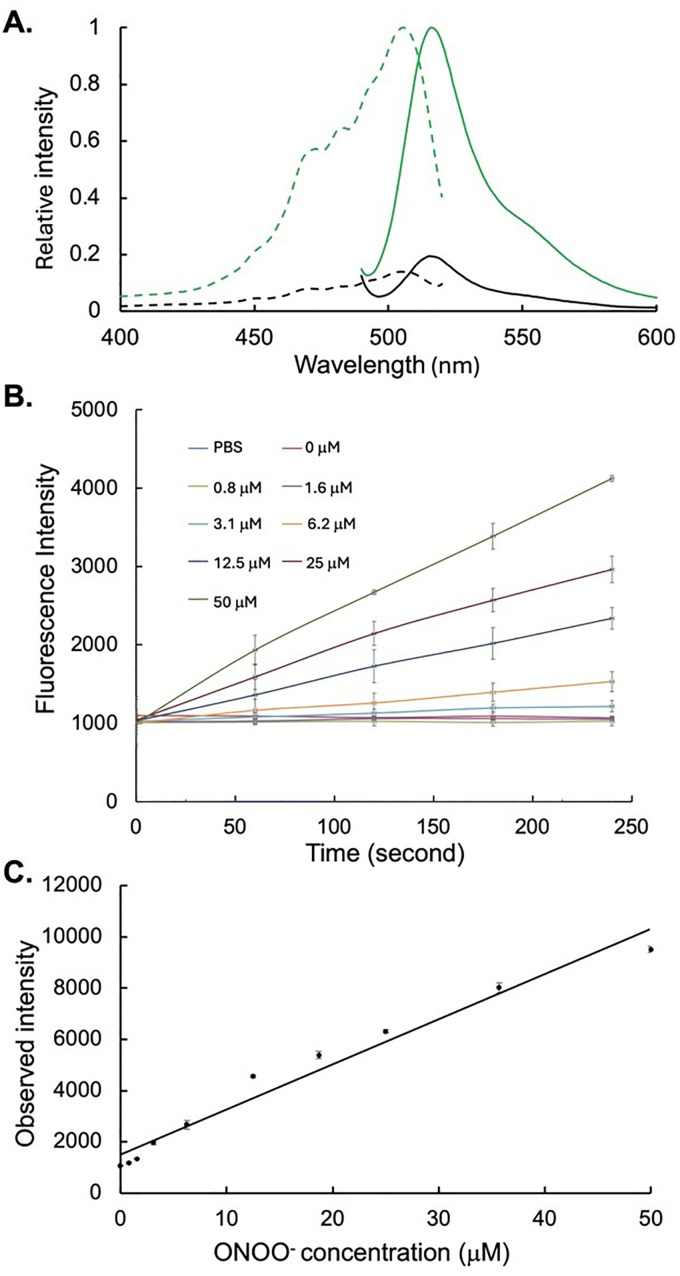
Fluorescence activation of cpmEos2-Y155BoPhe in response to peroxynitrite. (A) Fluorescence excitation (dashed lines) and emission (solid lines) spectra of boronic acid-containing cpmEos2 before (black) and after (green) 10 min incubation with peroxynitrite. Spectra were recorded after incubation of 200 µL protein (3.9 µM) with 50 µL of peroxynitrite (50 µM) at room temperature. (B) Time-dependent fluorescence response of cpmEos2-Y155BoPhe upon exposure to increasing concentrations of peroxynitrite (0 to 50 µM), showing rapid and concentration-dependent signal enhancement. (C) Concentration-dependent fluorescence response of cpmEos2-Y155BoPhe after 10 min of incubation with increasing concentrations of peroxynitrite. All measurements were performed at *λ*_ex_ = 485 nm and *λ*_em_ = 530 nm.

We next further characterized the sensing kinetics using a set of different concentrations of ONOO^−^. As shown in Fig. 2B, the sensor exhibited rapid and concentration-dependent fluorescence activation upon exposure to ONOO^−^, with signal increases observable within seconds of analyte addition. Higher concentrations of ONOO^−^ resulted in faster activation rates and greater fluorescence enhancement. Overall, the response was concentration-dependent over high nanomolar to low micromolar ranges, yielding a linear calibration curve (*R*^2^ = 0.9695; Fig. 2C). The limit of detection was 0.7 µM and the limit of quantification 2.1 µM at 3.9 µM protein concentration.

In contrast, H_2_O_2_ produced much lower fluorescence enhancement within short timeframes, consistent with literature kinetics showing ONOO^−^ reacts with boronates several orders of magnitude faster than H_2_O_2_.^17^ A comprehensive chemoselectivity panel demonstrated minimal response to HOCl, superoxide, hydroxyl radical, *tert*-butyl hydroperoxide, cysteine, ascorbate and NaHS under identical conditions, while ONOO^−^ induced robust activation (Fig. 3A). We also demonstrated that cpmEos2-Y155BoPhe can be activated by SIN-1,^18^ which is a peroxynitrite-generating donor (Fig. 3B). SIN-1 releases ^•^NO and O_2_^•−^*in situ*, which leads to their reaction and formation of peroxynitrite. This result confirmed the sensor's responsiveness under a continuous and graduate ONOO^−^ formation conditions. While SIN-1 activation proceeded more slowly than direct ONOO^−^ addition (SI, Fig. S6), a clear concentration-dependent response was observed (Fig. 3B).

**Fig. 3:**
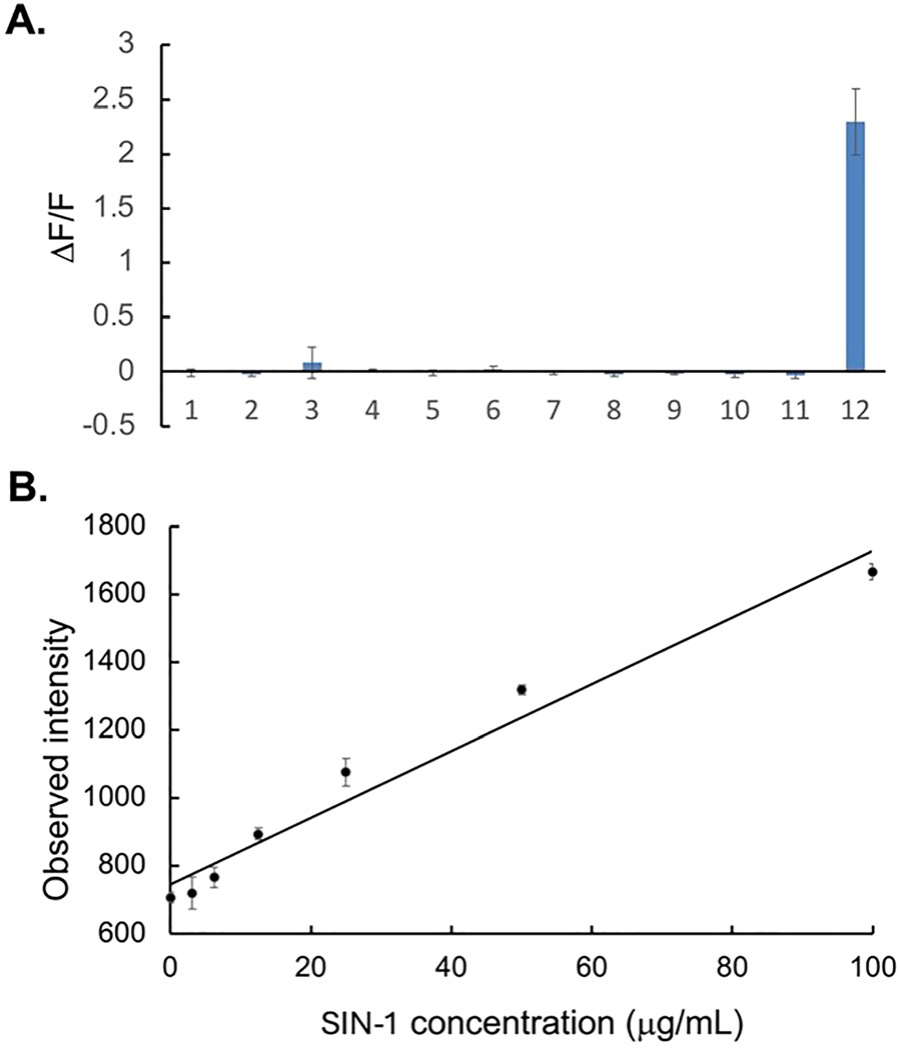
Chemoselectivity and *in situ* peroxynitrite detection using cpmEos2-Y155BoPhe. (A) Chemoselectivity of cpmEos2-Y155BoPhe. The response of purified biosensor (3.88 µM) was measured after incubation with various redox-active species for 15 min: (1) phosphate buffer, (2) 100 µM HOCl, (3) 100 µM NaHS (H_2_S donor), (4) 100 µM O_2_^•−^, (5) 1 mM l-ascorbic acid, (6) 5 mM l-cysteine, (7) 100 µM *tert*-butyl hydroperoxide (HOOtBu), (8) *tert*-butoxyl radical (^•^OtBu; generated from 1 mM Fe^2+^ and 100 µM HOOtBu), (9) hydroxyl radical (^•^OH; generated from 1 mM Fe^2+^ and 100 µM H_2_O_2_), (10) 100 µM H_2_O_2_, (11) 1 mM H_2_O_2_, and (12) 100 µM ONOO^−^. Samples were incubated at room temperature. (B) Calibration curve showing the linear relationship between SIN-1 concentration and fluorescence intensity after 10 min of incubation, demonstrating sensor activation by peroxynitrite generated *in situ* from SIN-1.

Importantly, peroxynitrite-activated cpmEos2-Y155BoPhe retained photoconversion capability. Following incubation with 50 µM peroxynitrite, 405 nm irradiation produced a 5.4-fold increase in red fluorescence (*λ*_ex_ = 560 nm; *λ*_em_ = 580 nm) between the 15 min irradiation sample (dark red curve) and the non-irradiated sample (gray curve, Fig. 4). The gray curve corresponds to ONOO^−^-activated cpmEos2-Y155BoPhe prior to photoconversion and exhibits minimal fluorescence near the emission maximum of the red species. Furthermore, extended irradiation increased the fraction of photoconverted chromophores, indicating that boronate oxidation restores canonical chromophore chemistry compatible with β-elimination and π-extension characteristic of Kaede-like fluorescence proteins.^19^ Compared to wild-type cpmEos2, cpmEos2-Y155BoPhe exhibited much reduced photoconversion efficiency (approximately 35% based on the fluorescence intensity; SI, Fig. S7). A small reduction in conversion efficiency is partly expected, as oxidation of cpmEos2-Y155BoPhe by peroxynitrite is incomplete under a short incubation (SI, Fig. S3). This resulted in a mixed population of converted and unconverted species. Nevertheless, the 65% lowered conversion efficiency suggest that additional factors may affect the photoconversion, such as the perturbation of the chromophore environment or the flexibility of the barrel due to peroxynitrite oxidation. While further optimization of the sensor is required for any real applications, this work demonstrates that genetically encoded reactive chemical functionality can be incorporated into a photoswitchable fluorescent protein without abolishing optical switching.

**Fig. 4:**
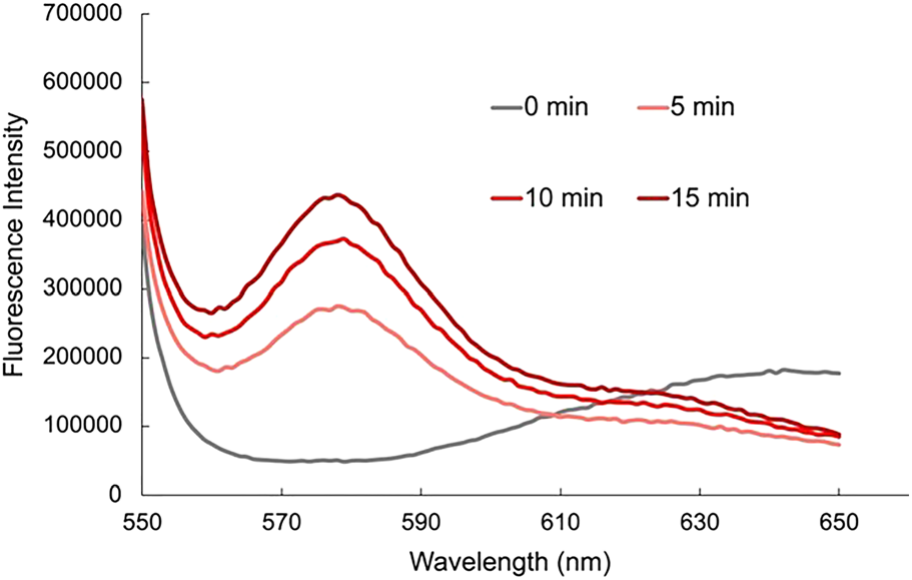
Photoconversion of peroxynitrite-activated cpmEos2-Y155BoPhe. Emission spectra (*λ*_ex_ = 560 nm; *λ*_em_ = 580 nm) of cpmEos2-Y155BoPhe (3.9 µM) before (gray) and after (red) photoconversion upon irradiation with 405 nm light. The 200 µL sensor sample was first activated by incubation with 50 µL ONOO^−^ (50 µM) for 15 min at room temperature prior to photoconversion. The gray curve corresponds to the emission spectrum (*λ*_ex_ = 560 nm; *λ*_em_ = 580 nm) of ONOO^−^-activated cpmEos2-Y155BoPhe prior to 405 nm irradiation. Progressive increases in red fluorescence were observed with increasing irradiation time (0 to 15 min), indicating successful photoconversion of the activated chromophore to the red-emitting Kaede-like form.

## Conclusions

We report a ncAA-containing photoconvertible fluorescent protein sensor for peroxynitrite detection. More broadly, the integration of ncAA-derived chemical reactivity into photoconvertible fluorescent protein scaffolds provides a strategy for engineering optically controllable biosensors. While the activated sensor does not fully recover the photophysical properties of wild-type cpmEos2, likely due to changes in the local chromophore environment following peroxynitrite treatment, substantial fluorescence activation and preserved photoconversion were nevertheless observed. The modular approach described here should be adaptable to other electrophilic or redox-responsive ncAAs, enabling broader integration of synthetic chemistry into advanced optical protein platforms. With further optimization to improve photoconversion efficiency and chromophore stability, future studies will explore application of this design in mammalian systems and advanced imaging modalities.

## Author contributions

G. M. Roman Arocho contributed to investigation, methodology, formal analysis, and writing (original draft). P. Das, S. Tennakoon, and J. B. Shaw contributed to methodology and mass spectrometric analysis. W. Niu and J. Guo contributed to conceptualization, project administration, supervision, and writing (review and editing).

## Conflicts of interest

There are no conflicts to declare.

## Supplementary Material

CB-007-D6CB00083E-s001

## Data Availability

All data supporting findings in this article are available within the article and in its online supplementary information (SI). Supplementary information is available. See DOI: https://doi.org/10.1039/d6cb00083e.
