# Supplementary material for: Engineering a light-controllable fluorescent protein for peroxynitrite detection via genetic code expansion
Source: RSC Chem Biol. 2026 Jun 18. Online ahead of print. doi: 10.1039/d6cb00083e (PMC13306567; doi:10.1039/d6cb00083e)
Supplement: CB-OLF-D6CB00083E-s001 [file CB-OLF-D6CB00083E-s001.pdf]

Supplementary Information (SI) for RSC Chemical Biology.
This journal is © The Royal Society of Chemistry 2026

Supplementary information

General Methods and Materials

Standard molecular biology techniques were used for plasmid construction and protein engineering.[1] DNA amplification was performed using Phusion High-Fidelity DNA Polymerase (New England Biolabs). Restriction enzymes were obtained from New England Biolabs and used according to the manufacturer's instructions. DNA sequencing was performed by Eurofins Genomics to confirm all constructs. Protein samples were analyzed by sodium dodecyl sulfate–polyacrylamide gel electrophoresis (SDS-PAGE) using 15% polyacrylamide gels prepared in-house and run on a Bio-Rad Mini-PROTEAN electrophoresis system. Precision Plus Protein Dual Color Standards (Bio-Rad) were used as molecular weight markers. Gels were stained with Coomassie Brilliant Blue and imaged using a Bio-Rad ChemiDoc XRS+ imaging system. Western blot analysis was performed using an anti-His6 tag antibody (Bio-Rad) and the Bio-Rad Immun-Blot Opti-4CN Colorimetric Detection Kit following the manufacturer's protocol. Fluorescence measurements were performed using a BioTek Synergy H1 Hybrid Multimode Microplate Reader and a Horiba FluoroMax spectrofluorometer. Absorbance measurements were obtained using a NanoDrop spectrophotometer (Thermo Scientific).

Plasmid Construction. The plasmid encoding cpmEos2, pAAV_hsyn_NES-his-CaMPARI2-WPRE-SV40, was a gift from Eric Schreiter (Addgene plasmid # 101060 ; http://n2t.net/addgene:101060 ; RRID:Addgene_101060)

pLei-cpmEos2-Y155TAG-MjytRNA. The chromophore-forming residue Y155 of cpmEos2 was mutated to an amber codon (TAG) using overlapping PCR. The PCR fragment was inserted into a pLei vector digested with NdeI and BplI using Sequence and Ligation Independent Cloning (SLIC). All plasmids were verified by DNA sequencing.

Protein Expression and Purification. The plasmid pBK-BoPheRS,[2] encoding the *p*-borono-L-phenylalanine (BoPhe)-specific aminoacyl-tRNA synthetase, was co-transformed into *E. coli* GeneHogs cells together with either pLei-cpmEos2-Y155TAG-mjYtRNA or pLei-mEos3.2-Y64TAG-mjYtRNA. Transformants were selected on LB agar plates containing kanamycin (50 μg/mL) and chloramphenicol (34 μg/mL). A single colony was inoculated into 5 mL LB medium containing the same antibiotics and grown overnight at 37 °C with shaking (250 rpm). The overnight culture was diluted 1:1000 into 10 mL LB medium containing antibiotics and grown at 37 °C until OD600 ≈ 0.6. Protein expression was induced by adding IPTG (0.5 mM) and BoPhe (1 mM). Cells were incubated for 15 h at 37 °C with shaking (250 rpm). Cells were harvested by centrifugation at 5000 g for 15 min at 4 °C and resuspended in lysis buffer (50 mM Tris-HCl pH 8.0, 300 mM NaCl, 10 mM imidazole). Cell lysis was performed either using B-PER reagent (Thermo Scientific) following the manufacturer's instructions or by sonication on ice (10 s on / 20 s off cycles for 5 min total). Cell debris was removed by centrifugation at 21,000 g for 30 min at 4 °C. The clarified lysate was applied to Ni-Sepharose 6 Fast Flow resin (GE Healthcare) and purified according to the manufacturer's protocol. Bound proteins were washed with 50 mM imidazole buffer and eluted using 250 mM imidazole. Purified proteins were buffer-exchanged into storage buffer (20 mM Tris-HCl pH 7.5, 150 mM NaCl, 10% glycerol) using Econo-Pac desalting columns (Bio-Rad) and Amicon Ultra-4 centrifugal filters (Millipore). Protein concentrations were determined using the Bradford protein assay (Bio-Rad). Purified proteins were aliquoted, flash-frozen in liquid nitrogen, and stored at -80 °C. Typical protein concentrations ranged from 180 to 430 μM.

Spectroscopic characterization. Fluorescence spectra were recorded using a Horiba FluoroMax spectrofluorometer. For $ONOO^-$-activated protein, excitation spectra were recorded from 400-505 nm with emission fixed at 516 nm. Emission spectra were recorded with excitation

fixed at 485 nm. For single-point measurements, fluorescence intensity was monitored at λex = 485 nm and λem = 516 nm. Photoconversion was induced after $ONOO^-$ activation by irradiation with 405 nm light for 15 cycles of 60 s irradiation followed by 30 s cooling intervals, corresponding to a total irradiation time of 15 min. The excitation slit width was set to 20 nm. After photoconversion, emission spectra were recorded with excitation at 545 nm. Fluorescence response was reported as dynamic range, calculated as:

$$(F_f - F_i)/F_i$$

where F_i represents the initial fluorescence intensity and F_f the final fluorescence intensity after $ONOO^-$ treatment.

Characterization of peroxynitrite Biosensor. Fluorescence were measured using a BioTek Synergy H1 Hybrid plate reader. Samples were maintained at 26 °C with orbital shaking for 2 s prior to each measurement. Fluorescence intensity was recorded every 60 s. For in vitro measurements, 200 μL protein solution (3.9 μM) was mixed with 50 μL diluted $ONOO^-$ stock solution to achieve the desired final concentration. $ONOO^-$ stock solutions were freshly prepared in 0.3 M NaOH, diluted to desirable concentrations, and adjusted pH to 7.4 before use.

LOD, LOL, and LOQ Calculations. The limit of detection (LOD) and limit of quantification (LOQ) were calculated according to International Conference on Harmonisation (ICH) guidelines.

$$LOD = 3.3 * (\frac{sd}{S})$$ and $$LOQ = 10 * (\frac{sd}{S})$$

Where *sd* is the standard deviation of blank measurements (n = 5) and *S* is the slope of the calibration curve (n = 3).

Sensor Selectivity Assay. Selectivity assays were performed using a BioTek Synergy H1 plate reader. A 50 μL solution of each reactive species was mixed with 200 μL of protein sensor (3.88 μM). Fluorescence was recorded every 30 s for 15 min at 26 °C. The following species were tested: HOCl (100 μM), NaHS (100 μM), superoxide (100 μM), L-ascorbic acid (1 mM), L-cysteine (5 mM), tert-butyl hydroperoxide (100 μM), hydroxyl radical (generated via Fenton reaction), hydrogen peroxide (100 μM or 1 mM), peroxynitrite (100 μM). Hydroxyl radicals were generated using Fe^{2+}/H_2O_2 (Fenton reaction), while tert-butoxyl radicals were generated using Fe^{2+}/HOOtBu. Peroxynitrite stock solutions were freshly prepared in 0.3 M NaOH, diluted to desirable concentrations, and adjusted pH to 7.4 before use. Their concentrations were determined spectrophotometrically at 302 nm (ε = 1670 M^{-1} cm^{-1}). Fluorescence responses were calculated as dynamic range $(F_f - F_i)/F_i$. All measurements were performed in triplicate.

Mass analysis. The denatured mass analyses were performed in positive ion mode on an Orbitrap Exploris 480 mass spectrometer (Thermo Fisher Scientific, Bremen, Germany). Both the non-oxidized and oxidized protein samples were prepared as 2 μM stock solutions (100 μL each). 50 μL from each stock solution was buffer exchanged into LCMS grade water with 0.1% formic acid (Fisher Scientific, Waltham, MA) using Amicon Ultra 0.5 mL 10 kDa molecular weight cutoff filters (Millipore Sigma, St. Louis, MO) following manufacturer's protocol. The pH of the solution was measured to be 2.45. Then, the desalted samples were diluted with 50:50 (v/v) acetonitrile/water with 0.1% formic acid (Fisher Scientific, Waltham, MA) to a final working concentration of 0.22 μM. Samples were then introduced into the instrument via direct infusion method at a flow rate of 5 μL /minute using electrospray ionization. The spray voltage was set at 3.4 kV at an ion transfer tube temperature of 320 °C. The MS1 for the non-oxidized sample was

acquired at a resolution of 120,000 and the oxidized sample at 240,000. The scan range for MS was set to 600-1500 m/z, and 100 microscans were averaged per each acquired spectrum.

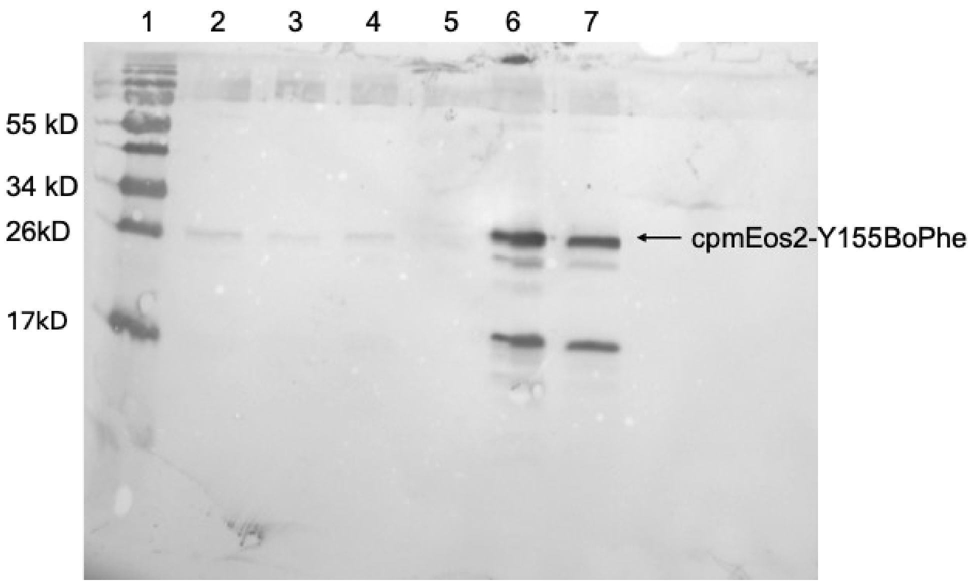

Fig. 1 Western blot analysis of cpmEos2-Y155BoPhe expression. Protein expression levels were analyzed under different induction and incorporation conditions. Lane 1, molecular weight marker; lane 2, soluble fraction without IPTG and without BoPhe; lane 3, total fraction without IPTG and without BoPhe; lane 4, soluble fraction with IPTG and without BoPhe; lane 5, total fraction with IPTG and without BoPhe; lane 6, soluble fraction with IPTG and with BoPhe; lane 7, total fraction with IPTG and with BoPhe. The band corresponding to cpmEos2-Y155BoPhe (~26 kDa) is indicated.

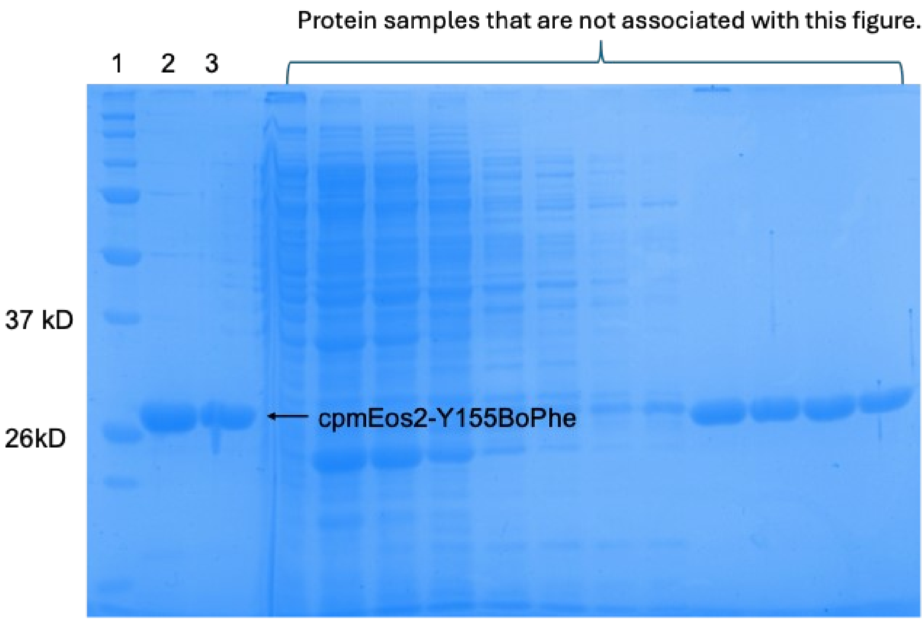

Fig. 2 SDS-PAGE analysis of purified cpmEos2-Y155BoPhe. Lane 1, molecular weight marker; lane 2, purified protein fraction 1; lane 3, purified protein fraction 2. The band corresponding to cpmEos2-Y155BoPhe (~26 kDa) is indicated.

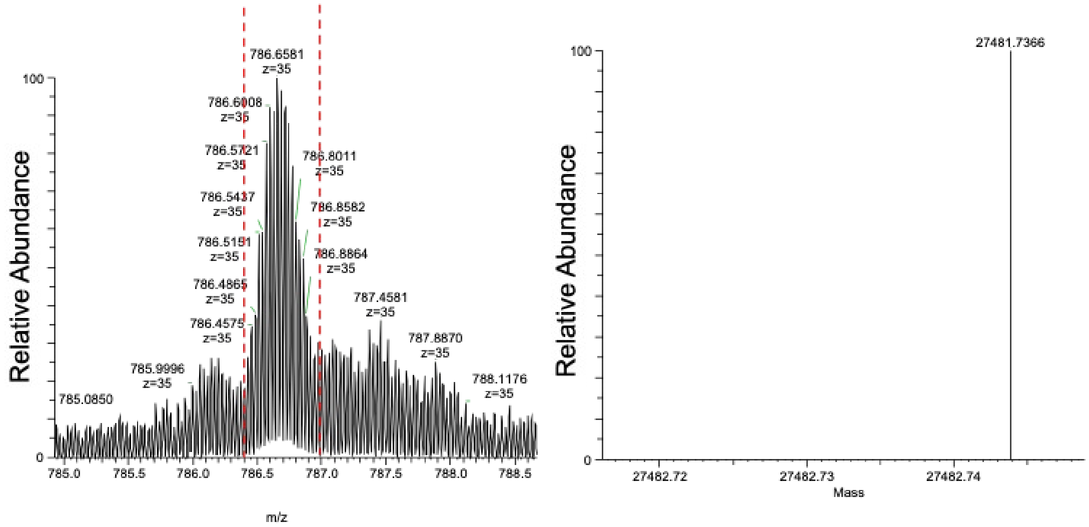

Fig. 3 Analysis of cpmEos2-Y155BoPhe by mass spectrometry. The observed mass is 27481.7366. The calculated mass is 27481.5170 (after loss of the N-terminal methionine).

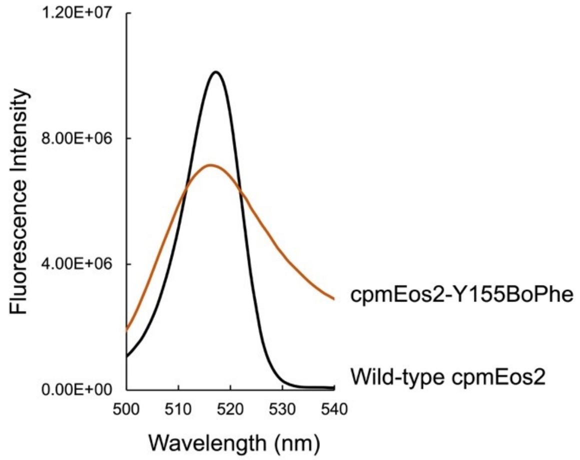

Fig. 4 Fluorescence emission spectra of peroxynitrite-treated cpmEos2-Y155BoPhe compared to wild-type cpmEos2. Emission spectra of wild-type cpmEos2 (black) and cpmEos2-Y155BoPhe (orange) after incubation with peroxynitrite (50 μM) at room temperature. Both samples were prepared at the same volume (250 μL) and protein concentration (3.9 μM).

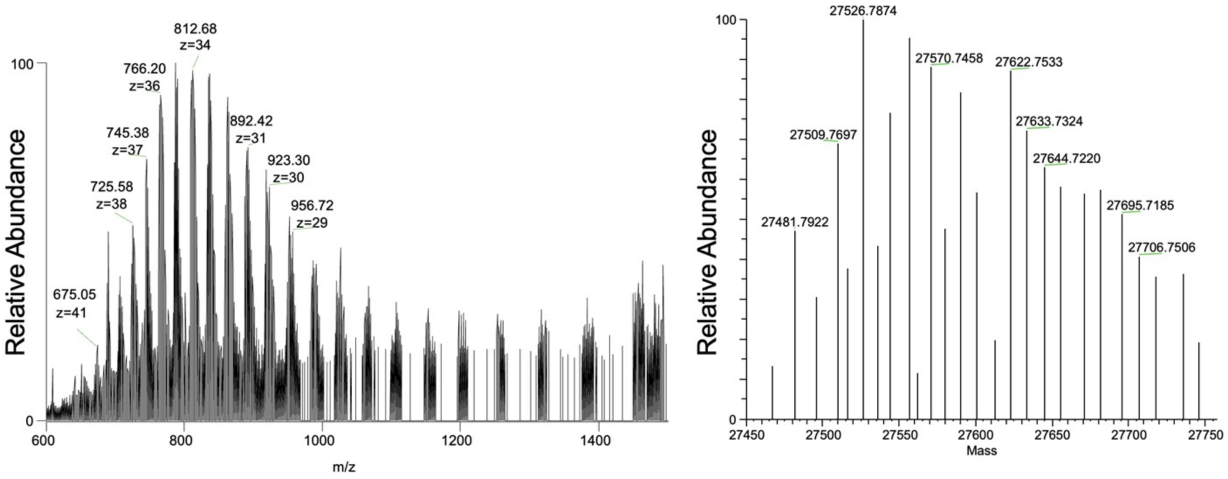

Fig. 5 Analysis of peroxynitrite oxidation of cpmEos2-Y155BoPhe by mass spectrometry. The calculated mass is 27453.50 (after loss of the N-terminal methionine). The detected masses correspond to different extents of oxidation of the three cysteine and eight methionine residues in the protein following peroxynitrite treatment.

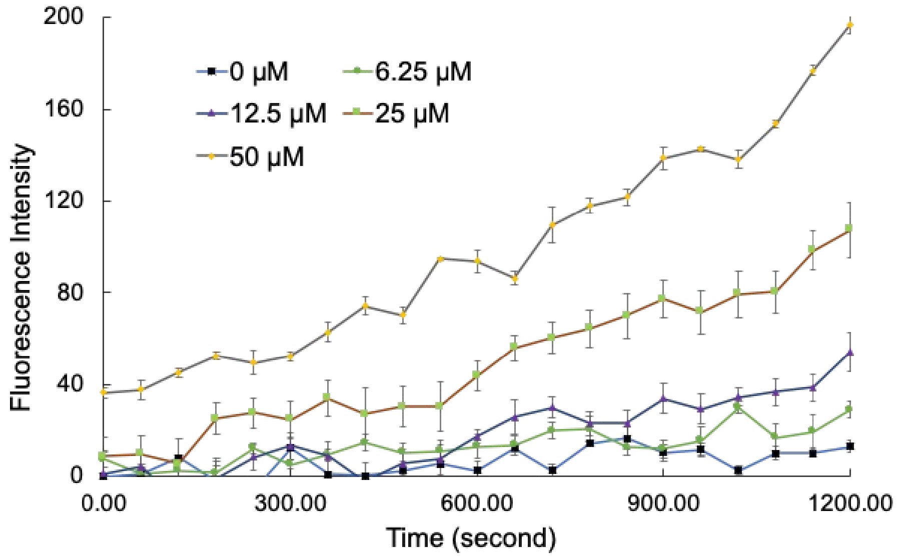

Fig. 6 Time-dependent fluorescence response of cpmEos2-Y155BoPhe upon exposure to increasing concentrations of SIN-1(0 to 50 μM), showing time- and concentration-dependent signal enhancement.

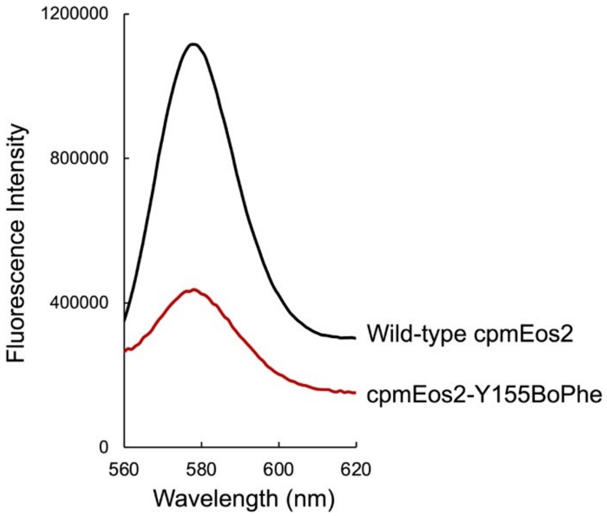

Fig. 7 Photoconversion efficiency of cpmEos2-Y155BoPhe compared to wild-type cpmEos2. Emission spectra of wild-type cpmEos2 (black) and cpmEos2-Y155BoPhe (red) after photoconversion under identical conditions. Both proteins were prepared at the same concentration (3.88 μM) prior to $ONOO^-$ treatment and 405 nm irradiation. The reduced fluorescence intensity of cpmEos2-Y155BoPhe reflects lower photoconversion efficiency relative to the wild-type protein.

References

(1) *Molecular cloning: A laboratory manual, third edition*; Cold Spring Harbor Laboratory Press, 2000.

(2) Brustad, E.; Bushey, M. L.; Lee, J. W.; Groff, D.; Liu, W.; Schultz, P. G. A genetically encoded boronate-containing amino acid. *Angew. Chem., Int. Ed.* **2008**, *47* (43), 8220-8223. DOI: 10.1002/anie.200803240.
